# First Principles Study on the Electronic Structure and Optical Properties of NCQDs/TiO_2_(101)

**DOI:** 10.3390/ma19143042

**Published:** 2026-07-15

**Authors:** Chao Luo, Jun Gao, Guoqing Gou, Kejun Jiang, Zhongyin Zhu

**Affiliations:** 1 CRRC Tangshan Co., Ltd., Tangshan 063000, China; 2 Key Laboratory of Advanced Technologies of Materials, Ministry of Education, School of Materials Science and Engineering, Southwest Jiaotong University, Chengdu 610031, China; 3 Southwest Jiaotong University Engineering Training Center, Chengdu 610031, China

**Keywords:** NCQDs, TiO_2_(101), first principles, electronic structure, optical properties

## Abstract

First-principles calculations based on density functional theory (DFT) were employed to investigate the binding energy, work function, electronic structure, and optical properties of carbon quantum dots (CQDs) and nitrogen-doped carbon quantum dots (NCQDs) loaded on the TiO_2_(101) surface. The results indicate that NCQDs exhibit stronger binding affinity to the TiO_2_(101) surface than CQDs. The charge transferred from CQDs to the TiO_2_(101) surface mainly originates from the carbon atoms located at the two ends along the a-axis of the CQDs. By contrast, the charge distribution between NCQDs and TiO_2_(101) is more uniform, with the Bader charges of the carbon atoms at the two ends along the a-axis in NCQDs being reduced to varying degrees compared to those in CQDs. Moreover, loading NCQDs is more favorable for the generation of photogenerated electron-hole pairs and electron transfer, whereas loading CQDs leads to the smallest band gap.

## 1. Introduction

Due to the increasingly severe energy crisis and environmental pollution, the demand for clean and sustainable energy is urgent. Among all renewable energy sources, hydrogen and solar energy are abundant resources that can respectively generate clean electricity through photovoltaic power generation and clean hydrogen via appropriate photocatalysts [1,2]. As a typical photocatalyst, titanium dioxide (TiO_2_) is widely applied due to its non-toxicity, good stability, and low cost. It exists in three crystalline forms: anatase, rutile, and brookite. Among these, anatase is widely recognized for its excellent photocatalytic performance. Fujishima and Honda [3] were the first to report water splitting driven by TiO_2_ photocatalysts; subsequently, water splitting research has expanded to semiconductors composed of transition metal oxides [4]. Although TiO_2_’s relatively wide band gap allows for sufficient utilization of ultraviolet light, it restricts its performance in the visible light region. Additionally, the high recombination rate of photogenerated electron-hole pairs poses a significant challenge.

In recent years, first-principles calculations have been widely employed to reveal the microscopic mechanisms underlying the photocatalytic performance of semiconductor materials. Fang and Luo et al. found that La and C co-doping in TiO_2_ introduces impurity levels through charge compensation between Ti 3d and C 2p states and forms a La_2_O_3_-TiO_2_ heterojunction, thereby reducing the band gap and suppressing carrier recombination [5,6]. Dongxiang Li et al. demonstrated that Sc/C co-doping on the TiO_2_(101) surface introduces shallow impurity states, achieving optimal visible-light absorption at a dopant concentration of 5.56% [7]. Wu and Zhu et al. pointed out that sub-bands derived from C 2p and Cr 3d states in Cr-C co-doped anatase TiO_2_ narrow the effective band gap to approximately 0.86–1.19 eV [8,9]. Furthermore, Zhan Qu et al. confirmed that a rational interfacial band alignment and built-in potential in BiOI/TiO_2_ heterojunctions significantly promote photogenerated carrier separation [10]. In summary, DFT calculations provide important theoretical foundations for understanding the modulation mechanisms of doping and heterojunctions on the electronic structure and photophysical properties of semiconductors.

Based on theoretical calculations, numerous regulation strategies have been developed to enhance the photoelectrochemical activity of TiO_2_ materials, such as single-doping or co-doping with metals/non-metals [11], organic dye sensitization [12], coupling with narrow band gap semiconductors [13], and stress–strain engineering [14], among others. Recently, quantum dots have attracted attention as a novel photocatalytic and photoelectronic material due to their quantum size effects [15]. Under illumination, quantum dots can generate numerous electron-hole pairs; thus, carbon quantum dots (CQDs) have been utilized as a new, efficient, and green sensitizer to enhance the photoelectrochemical performance (PEC) of TiO_2_. Zhou et al. [16] demonstrated that, compared with the pristine system, the CQD-modified A/R-TiO_2_ considerably improved visible light absorption, bulk charge separation efficiency, and surface charge injection efficiency. Saud et al. [17] prepared CQDs/TiO_2_ nanofibers via a simple one-step hydrothermal method and found that anchoring quantum dots not only enhanced light absorption but inhibited the recombination of photogenerated electron-hole pairs, thereby improving the catalytic and antibacterial performance. The catalytic activity of CQDs can be further enhanced by doping with different elements. These newly generated energy states shift the Fermi level toward the conduction band, resulting in faster electron exchange compared to the original CQDs [18,19]. Nitrogen atoms, being almost the same size as carbon atoms with five valence electrons available for bonding with other carbon atoms, increase the surface defect states of CQDs upon doping, thereby enhancing their optical and catalytic activities. TiO_2_ modified with nitrogen-doped carbon quantum dots (NCQDs) not only improves visible light absorption but facilitates electron transfer and the separation of photogenerated electron-hole pairs [20,21]. Although numerous experimental results have indicated that the modification of TiO_2_ with CQDs and their derivative materials can enhance its visible light absorption and suppress the recombination of photogenerated charge pairs, the interaction mechanism between CQDs (and their modified counterparts) and TiO_2_ remains unclear, impeding further improvement of quantum dot-modified TiO_2_ materials. Therefore, this paper employs first-principles calculations to investigate the electronic structure and optical properties of TiO_2_(101), CQDs/TiO_2_(101), and NCQDs/TiO_2_(101) in order to explore their microscopic interaction mechanisms.

## 2. Calculation Methods

**Figure 1:**
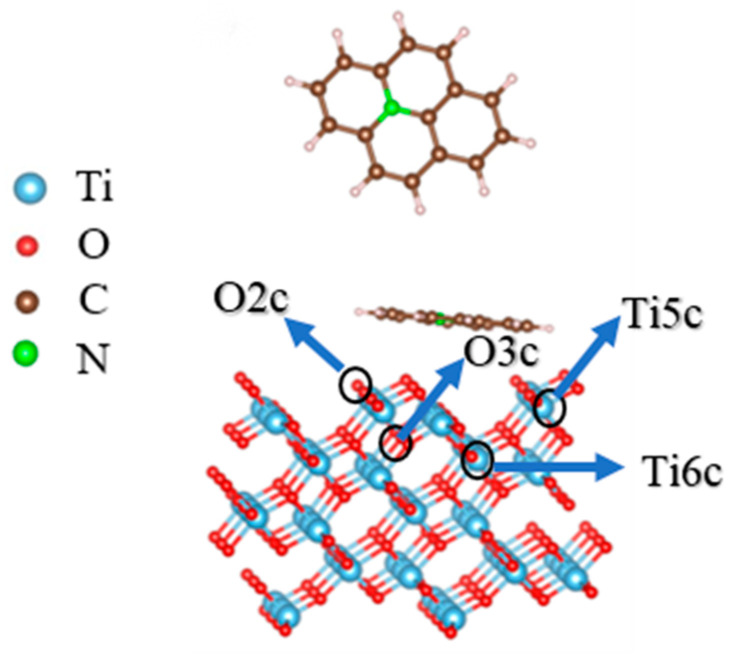
Optimized Surface Models for Different Architectures: NCQDs/TiO_2_(101).

In this study, the electronic structure and optical properties of NCQDs/TiO_2_(101) were calculated using the first-principles calculation software VASP.6.1.0 [22] (Vienna Ab-initio Simulation Package) based on density functional theory. The interactions between electrons and ions were accurately described employing the projector augmented-wave (PAW) method [23], while the exchange-correlation potential among electrons was treated using the Perdew–Burke–Ernzerhof (GGA-PBE) approach under the generalized gradient approximation. To account for the strong Coulomb repulsion among Ti 3d electrons in anatase TiO_2_, a Hubbard parameter (U) was incorporated into the GGA functional. Through U-value testing, the U value for Ti d electrons was set at 2.3 eV. A 3 × 3 × 1 supercell was constructed for the anatase TiO_2_(101) plane with a 10 Å vacuum layer established in the Z-direction; this surface is typically predicted to be the most stable. The surface model was built as a periodic structure consisting of 9 atomic layers, as shown in Figure 1. Due to the complex structure of CQDs in actual systems, accurate modeling of CQDs has remained a challenge. Carbon clusters or graphene quantum dots (GQDs) are often used as substitutes for CQDs in first-principles calculations [24]. In this work, GQDs were adopted as substitutes for CQDs to investigate the properties of various systems, as also illustrated in Figure 1. The plane-wave basis set cutoff energy was fixed at 450 eV, and the Brillouin zone of anatase TiO_2_ was sampled using a uniform 2 × 2 × 1 Monkhorst–Pack [25] k-point grid. The convergence criteria for the geometric optimization calculations were set at 10^−5^ eV/cell for energy and −0.01 eV/Å for forces.

## 3. Results and Discussion

### 3.1. Interface Coupling Parameters

Through DFT calculations, the atomic-level interactions between NCQDs and the TiO_2_(101) surface were investigated, revealing that carbon quantum dots in different systems are stretched upward in the Z direction. The minimum distance between the CQDs and the TiO_2_(101) surface is 3.32 Å, which is greater than the corresponding distance of 3.28 Å for NCQDs, indicating that the interaction between NCQDs and the TiO_2_(101) surface is stronger than that for CQDs. Furthermore, the binding energy between the quantum dots and the TiO_2_(101) surface was calculated, with the corresponding formula provided below:

$$E_{b}=E_{A}+E_{B}-E_{A/B}$$

where E_b_ represents the binding energy of the entire A/B system (in eV), E_A_ and E_B_ denote the total energies of individual A and B after relaxation, respectively, and E_A/B_ represents the total energy of the entire A/B system after relaxation.

Weak interactions were observed between the CQDs of different systems and TiO_2_(101). The binding energy of CQDs/TiO_2_(101) is 0.014 eV, and that of NCQDs/TiO_2_(101) is 0.756 eV, indicating that NCQDs are more likely to be loaded on the surface of TiO_2_(101).

The binding energy of NCQDs (0.756 eV) is much higher than that of CQDs (0.014 eV). This discrepancy mainly arises because conventional CQDs are decorated with abundant surface oxygen-containing functional groups (e.g., −OH and −COOH), which bind to TiO_2_ via esterification or condensation to form weak Ti−O−C linkages [26].

Apart from Ti−O−C bonding, NCQDs can construct pyrrolic N-related or O−Ti−N bonds with TiO_2_. Benefiting from plentiful amino and carboxyl groups on their surface as well as the intrinsic sp^2^-hybridized carbon skeleton, NCQDs are capable of generating π–π stacking interactions with surface −OH groups of TiO_2_ or target organic molecules. Such interactions optimize electronic level alignment to accelerate interfacial charge transfer, raise the density of surface functional groups and available active sites, and consequently facilitate the immobilization of NCQDs onto the TiO_2_ substrate.

### 3.2. Work Function and Electronic Structure

**Figure 2:**
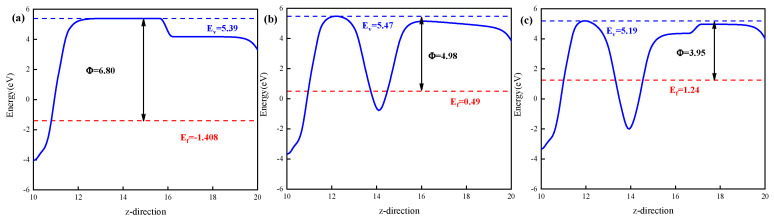
Work functions of different systems: (**a**) TiO_2_(101); (**b**) CQDs/TiO_2_(101); (**c**) NCQDs/TiO_2_(101).

The electronic structure and work function (Φ) of NCQDs/TiO_2_(101) determine their photoelectric performance; therefore, the work functions, band structures, and density of states of various systems were calculated using first principles. Figure 2 shows the work functions of the different systems. Compared with isolated TiO_2_(101), it is observed that the work function of the quantum dot-loaded TiO_2_ structure decreases, with NCQDs/TiO_2_(101) exhibiting the lowest work function (Φ = 3.95 eV). Previous studies [27,28,29] have indicated that composite nanomaterials show a significant reduction in work function compared to isolated materials. The reduction in the system’s work function caused by the synergistic electronic interactions between NCQDs and TiO_2_(101) is likely to promote the rapid generation of electron-hole pairs [30,31].

**Figure 3:**
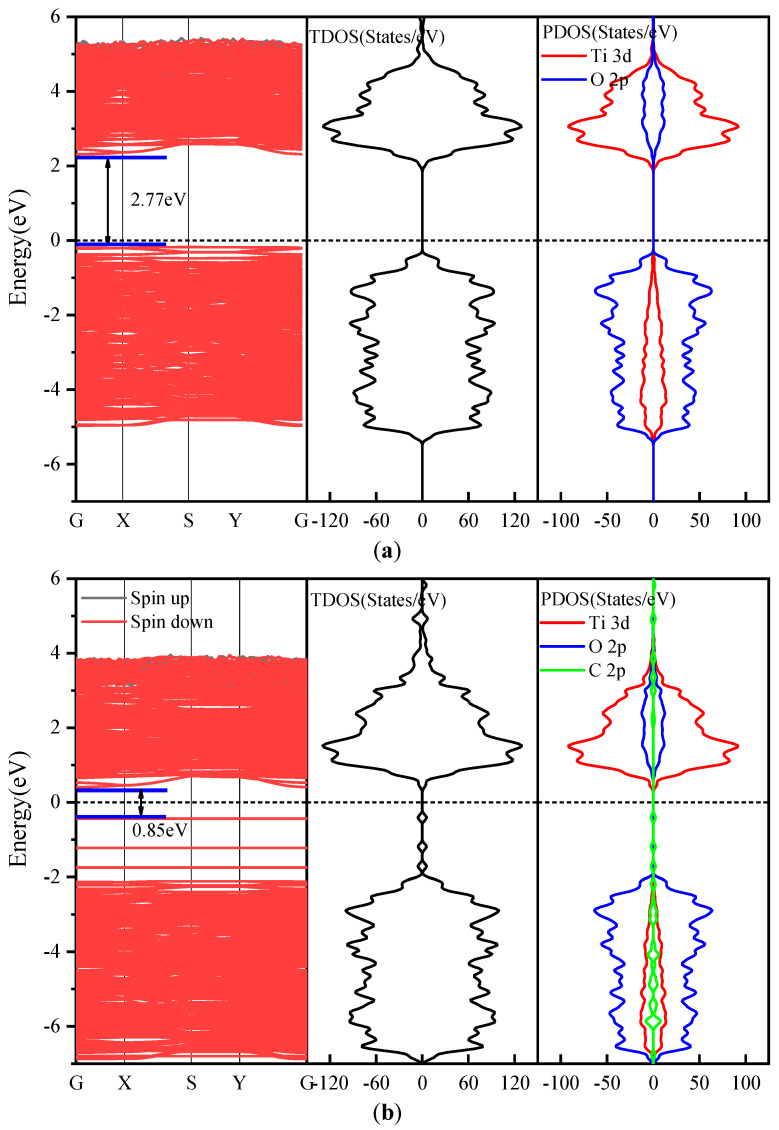

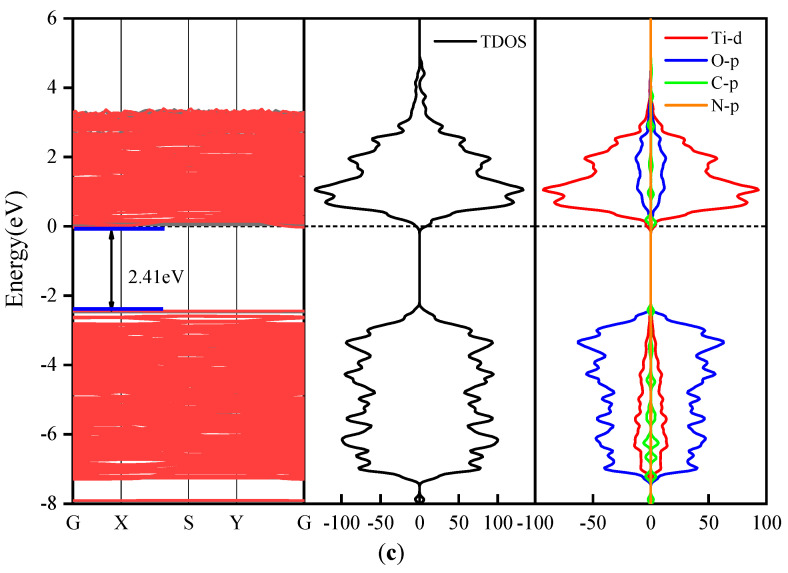
Band and density of states of (**a**) TiO_2_(101), (**b**) CQDs/TiO_2_(101), (**c**) NCQDs/TiO_2_(101).

Figure 3 illustrates the band structures and density of states for TiO_2_(101), CQDs/TiO_2_(101), and NCQDs/TiO_2_(101). As shown in Figure 3a, pure TiO_2_ possesses an indirect band gap with a value of 2.49 eV, which is lower than the experimental value of 3.2 eV [32,33]. This discrepancy arises from an inherent limitation of the PBE functional, as the Kohn–Sham equation in density functional theory does not account for the system’s excited states. Since this study focuses on the variation of band gaps among different systems, this issue does not affect our results. In pure TiO_2_, the valence band is mainly contributed by O 2p electrons, while the conduction band primarily originates from Ti 3d electrons, which is consistent with the calculations by other researchers [34]. After loading CQDs onto the surface of TiO_2_(101), three new donor levels were introduced below the Fermi level, resulting in a reduction of the band gap to 0.85 eV (see Figure 3b). Feng et al. [35] observed a reduction in the band gap to 2.77 eV in CQDs/TNRs synthesized via a hydrothermal method. Furthermore, absorption in the visible light region was significantly enhanced, owing to the fact that CQDs enable single photons to generate multiple excitons and play an important role in solar light utilization. A closer look at Figure 3b reveals that the new energy levels are mainly contributed by C 2p electrons, without hybridization with Ti and O atoms, indicating a weak interaction between CQDs and TiO_2_(101). Therefore, the enhancement in the visible light absorption of CQDs/TiO_2_(101) relies almost exclusively on the intrinsic properties of CQDs. Upon doping CQDs with nitrogen, the band gap of the system decreased slightly to 2.41 eV compared to that of pure TiO_2_(101) (see Figure 3c), while the Fermi level shifted upward to the bottom of the conduction band, thereby increasing the carrier mobility. This observation agrees with the computational results of Yashwanth et al. [31]. As shown in Figure 3c, the occupied C 2p orbitals enter the band gap of the TiO_2_ substrate, while the unoccupied orbitals extend into the conduction band of the substrate. This staggered band arrangement not only represents an important prerequisite for electron injection but facilitates the transition of electrons from the conduction band minimum of TiO_2_ to the HOMO of NCQDs. Although the reduction in the band gap is less pronounced compared to that observed with CQDs (i.e., the enhancement in visible light absorption is relatively modest), the introduction of nitrogen effectively eliminates the impurity levels generated by CQDs, considerably reducing the recombination of photogenerated electron-hole pairs and prolonging the lifetime of the photogenerated charge carriers. The TDOS integrates over all k-points in the Brillouin zone and counts the number of electronic states within a unit energy at all k-points. Even if a state exists only at k-points along non-high-symmetry paths, the TDOS will include it in the count, thus the TDOS is not zero.

The mobility of charge carriers depends on their effective mass, and the calculation formula is given as follows:

(1) $$m^{*}=\hbar {\left(\frac{d^{2}E}{dk^{2}}\right)}^{-1}$$

(2) $$\mu =\frac{q\tau }{m^{*}}$$

In the equation, E, k, and h represent the band energy (eV), wave vector, and Planck constant (6.626 × 10^−34^ J/s), respectively, and μ, q, and τ denote the carrier mobility (cm^2^/V/s), the charge (e), and the scattering time (s), respectively. By calculating the energy band fitting function of E versus k, the effective mass of electrons and holes in different systems were obtained, as shown in Table 1. Both the CQD and NCQD systems exhibit a lower effective mass compared to the pristine system, with NCQDs having the lowest carrier effective mass and a more pronounced reduction in the effective mass of photogenerated electrons. Equation (2) indicates that the lower the effective mass, the higher the mobility of the photogenerated carriers. Therefore, consistent with the density of state results, the NCQD system can provide more electrons, and there is a qualitative trend of improvement in the band-shaped transmission.

### 3.3. Charge Density

Charge density difference, also known as charge density disparity, is commonly referred to as CDD or DCD. It is a core tool used in first-principles calculations based on density functional theory for analyzing the redistribution of electrons. Its physical essence describes the changes in electron density that occur after specific processes, such as bonding, adsorption, doping, and interface recombination in the system. By comparing the charge density distribution of the target system with that of each isolated component, it quantitatively describes the aggregation and dissipation behavior of electrons. Its physical significance indicates that positive regions represent charge accumulation, i.e., electron enrichment, while negative regions represent charge dissipation, i.e., electron depletion.

**Figure 4:**
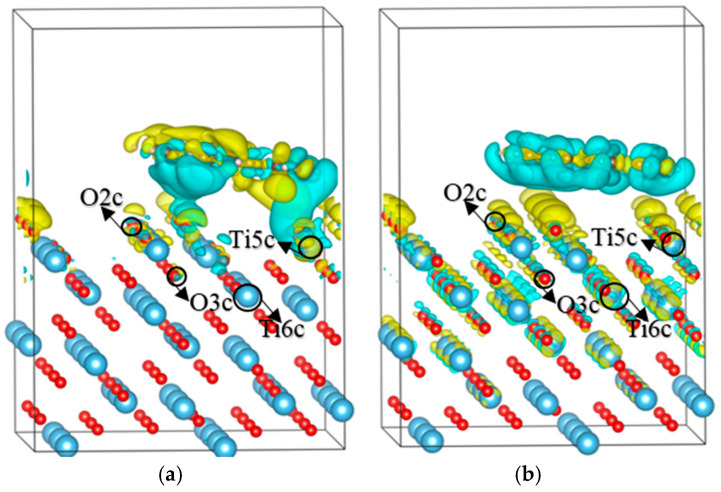
Differential charge densities of (**a**) CQDs/TiO_2_(101) and (**b**) of NCQDs/TiO_2_(101).

Differential charge density serves as a key method for determining whether a chemical bond is formed between two atoms, providing an intuitive means of observing interfacial charge transfer. Figure 4 displays the differential charge density of both the CQD/TiO_2_(101) and the NCQD/TiO_2_(101) systems. In the region of the most intimate interfacial interaction, an accumulation of electron density is observed (Figure 4), indicating that an electronic interaction between the NCQDs and the TiO_2_(101) surface has altered the electronic structure, shifting the Fermi level toward the conduction band edge. As shown in Figure 4a, charge transfer primarily occurs between the CQDs and the 2-coordinated O atoms (O2c), as well as the 5-coordinated Ti atoms (Ti5c) on the surface. By contrast, the electron distribution at the NCQDs/TiO_2_(101) interface is more uniform (Figure 4b), which is consistent with the computational results reported by other researchers [31].

To further analyze the charge characteristics of the structure, we calculated the Bader charges for different systems. In the case of the TiO_2_(101) surface, the Bader charges of the Ti atoms were determined to be +2.3 e for the Ti6c sites and +2.27 e for the Ti5c sites, while those of the surface oxygen atoms were −1.15 e for the O_3c_ sites and −0.89 e for the O_2c_ sites, in agreement with previous studies [36]. For the loaded CQDs, the C atoms at the two ends along the a-axis exhibit Bader charges of +0.2 e and +0.11 e, respectively, with the remaining C atoms showing charges close to 0. Thus, the charge transferred from the CQDs to the TiO_2_(101) surface mainly originates from the C atoms at the two ends along the a-axis, which is consistent with the differential charge density analysis. By contrast, for the loaded NCQDs, the N atoms have a Bader charge of −0.71 e, and the Bader charges of the C atoms at the a-axis ends are reduced to varying extents relative to those in the CQDs. Furthermore, the Bader charge of the O_2c_ atoms on the TiO_2_(101) surface, which are closest to NCQDs, increases by approximately 0.12 e compared to that of CQDs. Therefore, the loading of NCQDs more effectively facilitates electron transfer within the system.

### 3.4. Optical Properties

**Figure 5:**
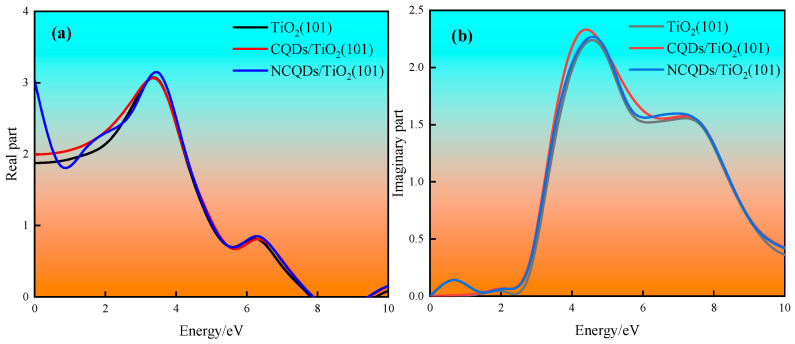
The complex dielectric functions of (**a**) Static dielectric function, (**b**) After loading with quantum dots.

The complex dielectric function, ε(ω) = ε_1_(ω) + iε_2_(ω), is used to describe the optical properties of semiconductors within the linear response regime. Figure 5 exhibits the complex dielectric functions for various systems. Regarding the real part of the static dielectric function (Figure 5a), the order is TiO_2_(101) < CQDs/TiO_2_(101) < NCQDs/TiO_2_(101), indicating that TiO_2_ loaded with NCQDs has an enhanced dielectric performance, which improves the internal polarization capacity and facilitates the migration and separation of photogenerated charge carriers. For pristine TiO_2_(101), the imaginary part begins to respond at 0.696 eV and reaches a peak at 4.87 eV, indicating that the original system has a relatively poor response within the visible light range of 1.64 eV to 3.19 eV. After loading with quantum dots, the peak shifts negatively, thereby enhancing the response to visible light (Figure 5b).

In VASP, simulating the optical properties of semiconductors involves calculating the variation of the dielectric function ε_2_ with frequency, and then deriving optical constants, such as the absorption coefficient and refractive index from this. VASP directly provides the starting position of ε_2_, which corresponds to the energy (direct transition energy) of the DFT electronic band gap. Using the Tauc plotting method, the “direct optical band gap” can be extrapolated. In most inorganic semiconductors, the values of the electronic band gap and the optical band gap are nearly identical at room temperature (as described in this article for the TiO_2_ material); while in organic semiconductors and two-dimensional materials, the electronic band gap is numerically equal to the optical band gap plus the exciton binding energy. The optical band gap calculated by DFT usually significantly underestimates the true value (PBE generally underestimates by 30–50%), because the Kohn–Sham energy gap is essentially the electronic band gap and is limited by the functional.

**Figure 6:**
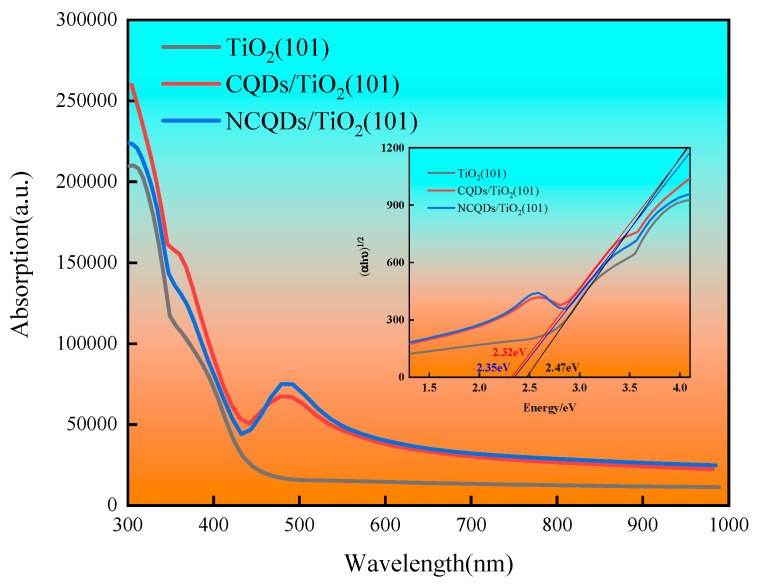
Absorption spectra and optical band gaps of TiO_2_(101), CQDs/TiO_2_(101), and NCQDs/TiO_2_(101).

The absorption spectra of TiO_2_(101), CQDs/TiO_2_(101), and NCQDs/TiO_2_(101) were calculated to investigate the influence of quantum dots on the optical properties of TiO_2_, as presented in Figure 6. Owing to the intrinsic bandgap of TiO_2_, all three systems exhibit strong ultraviolet absorption. Upon loading with quantum dots, a pronounced redshift of the absorption edge and an increase in absorption intensity in the visible region are observed, which agrees with experimental results [37]. A combined analysis of the band structure and density of states indicates that the loading of CQDs introduces three donor energy levels within the TiO_2_ bandgap, thereby reducing the energy required for electrons in the valence band to transition to the conduction band. Similarly, the loading of NCQDs shifts the system’s Fermi level upward toward the conduction band, also lowering the energy necessary for electron transitions.

## 4. Conclusions

In this study, we employed first-principles calculations to investigate the binding energy, work function, electronic structure, and optical properties of CQDs and NCQDs loaded on the TiO_2_(101) surface. The following conclusions are drawn:

(1) A weak interaction exists between CQDs and the TiO_2_(101) surface, with NCQDs binding to the TiO_2_(101) surface more readily than CQDs.
(2) The NCQD/TiO_2_(101) system exhibits the lowest work function, which is most beneficial for promoting the generation of photogenerated electron-hole pairs and electron migration. In addition, the CQD/TiO_2_(101) system introduces three new donor levels below the Fermi level, resulting in the smallest band gap (0.85 eV). After N-doping of the CQDs, the Fermi level shifts to the bottom of the conduction band; although the band gap increases, it remains smaller than that of TiO_2_(101), and there is a qualitative trend of improvement in the band-shaped transmission.
(3) In the CQD system, charge transfer primarily occurs between the two-coordinated oxygen atoms (O_2c_) at the surface and the five-coordinated titanium atoms (Ti_5c_); the charge transferred from the CQDs to the TiO_2_(101) surface mainly originates from the carbon atoms located at both ends along the a-axis. In the case of NCQDs, the charge distribution with the TiO_2_(101) surface is more uniform, and the Bader charges of the carbon atoms at both ends along the a-axis are reduced to varying extents compared to those in CQDs.
(4) The optical property calculations indicate that the abilities of photogenerated charge carrier migration, separation, and visible light response increase in the following order: TiO_2_(101) < CQDs/TiO_2_(101) < NCQDs/TiO_2_(101).

## Figures and Tables

**Table 1:** Effective Mass of Charge Carriers in Different Systems.

	TiO_2_(101)	CQDs/TiO_2_(101)	NCQDs/TiO_2_(101)
$m_{e}^{*}/m_{0}$	2.25	0.25	0.089
$m_{h}^{*}/m_{0}$	0.36	0.34	0.21

## Data Availability

The original contributions presented in this study are included in the article. Further inquiries can be directed to the corresponding author.
